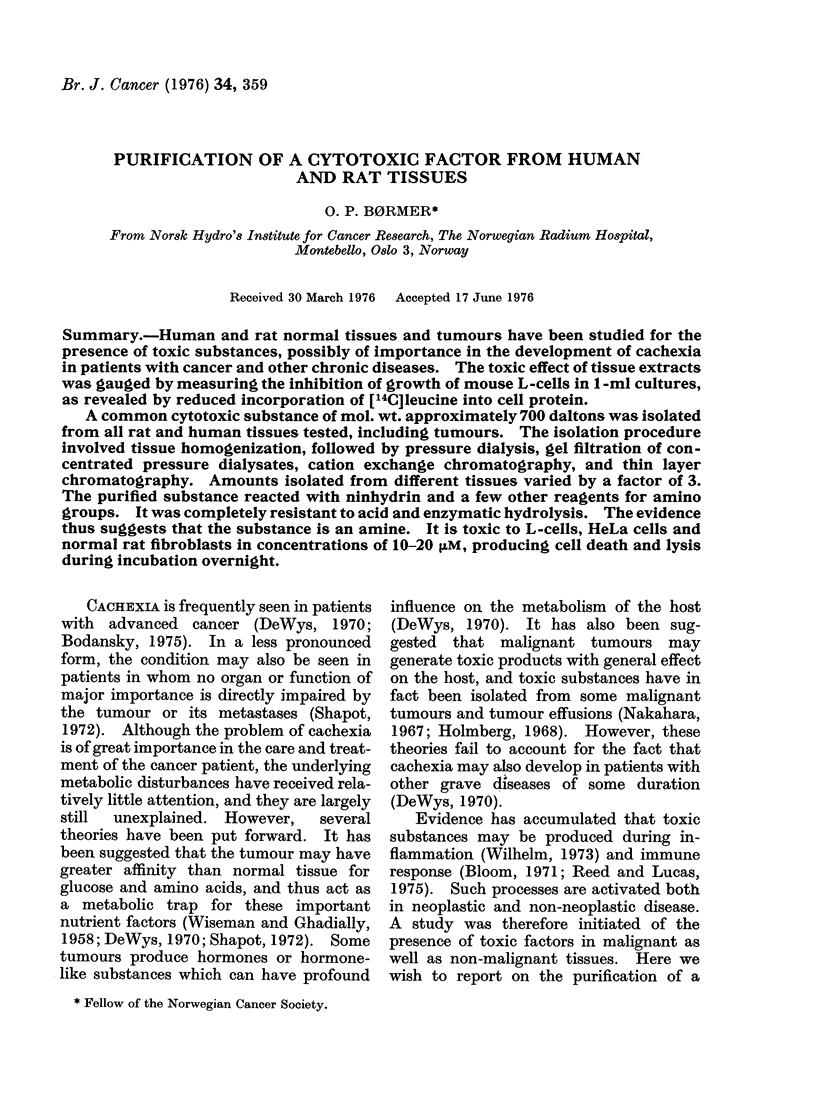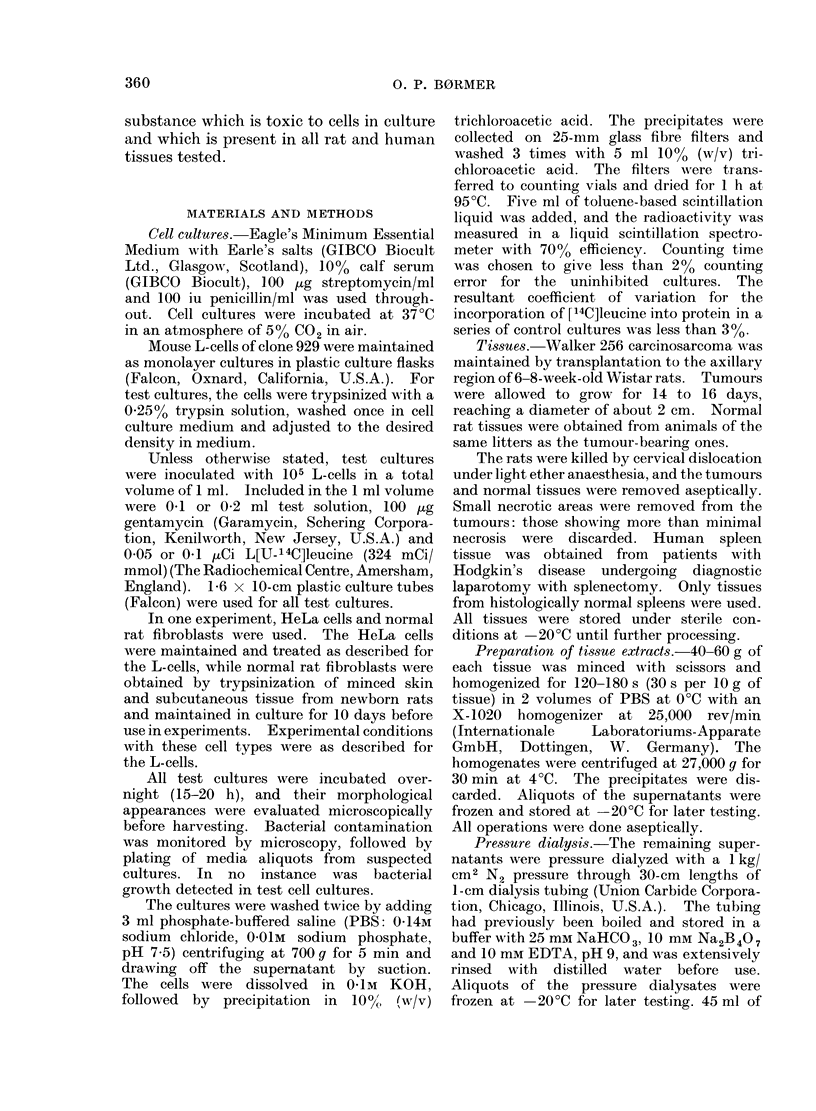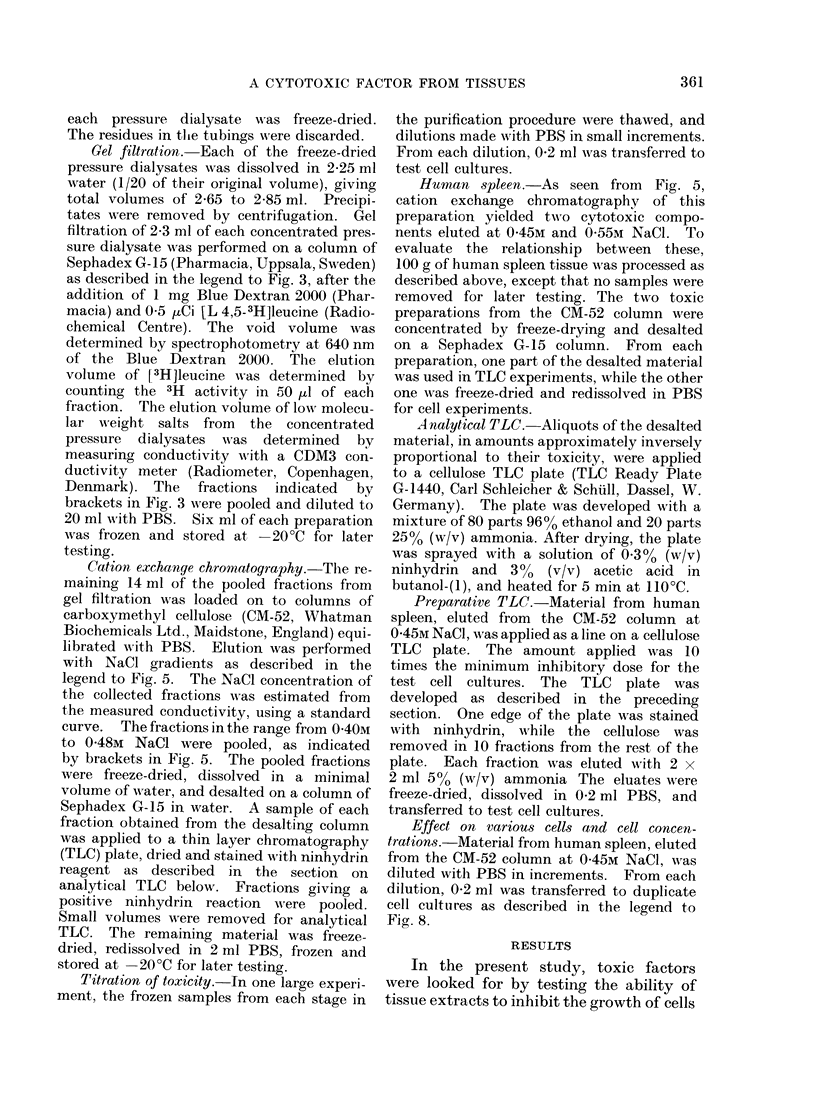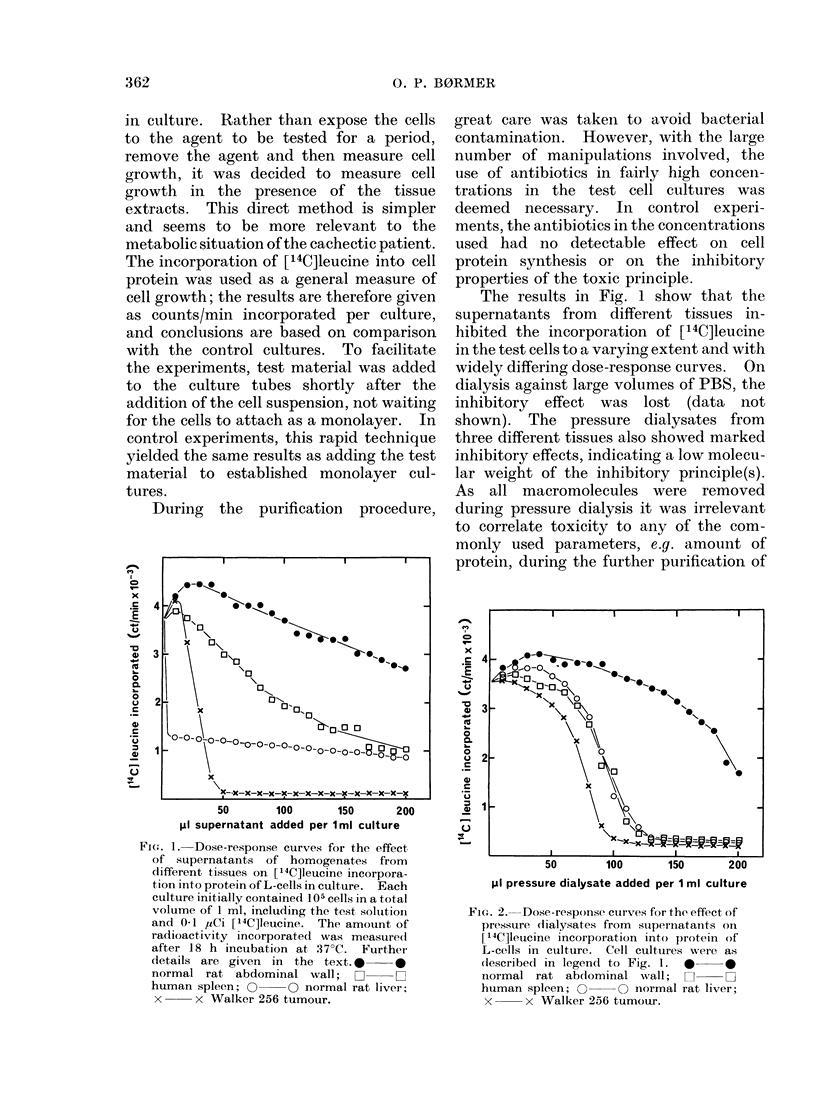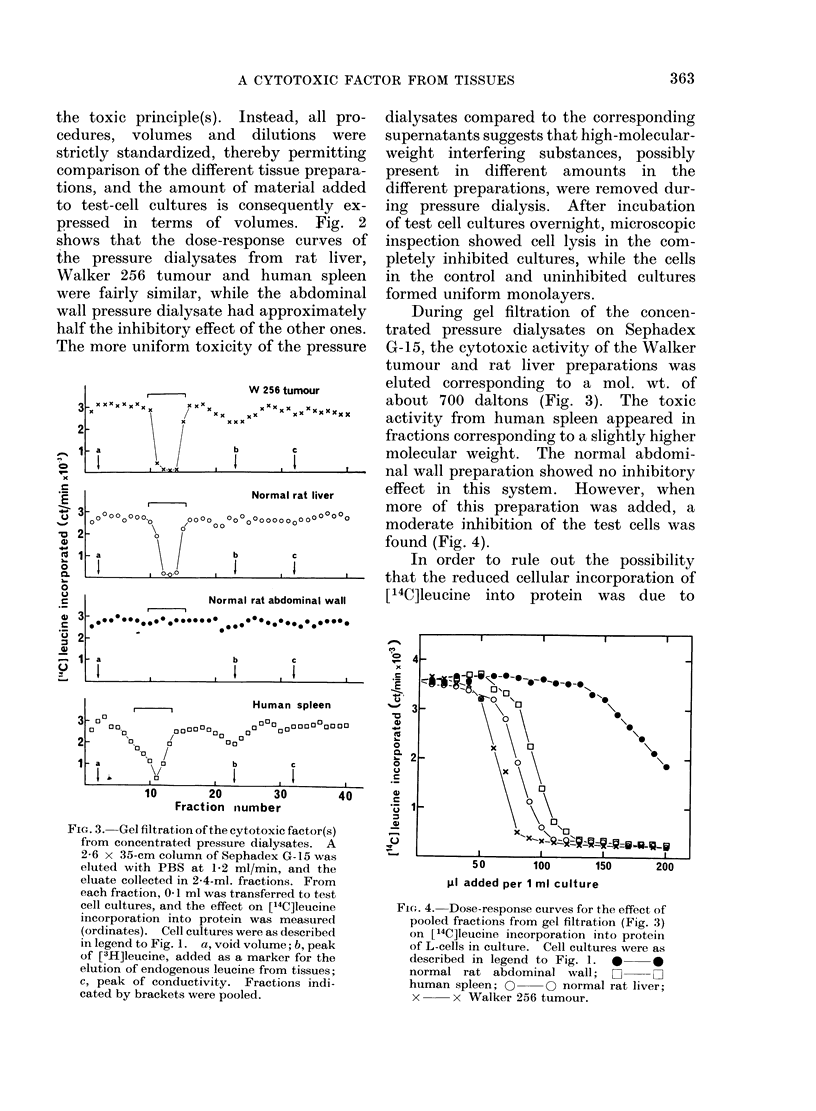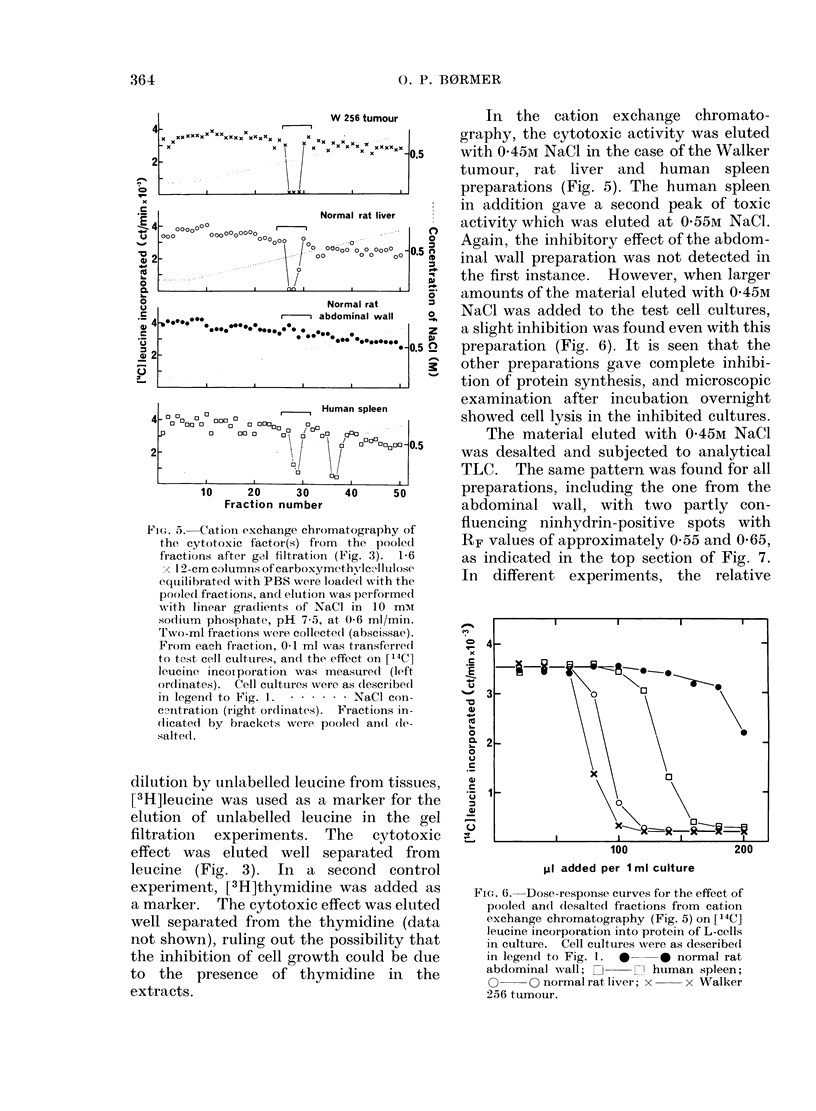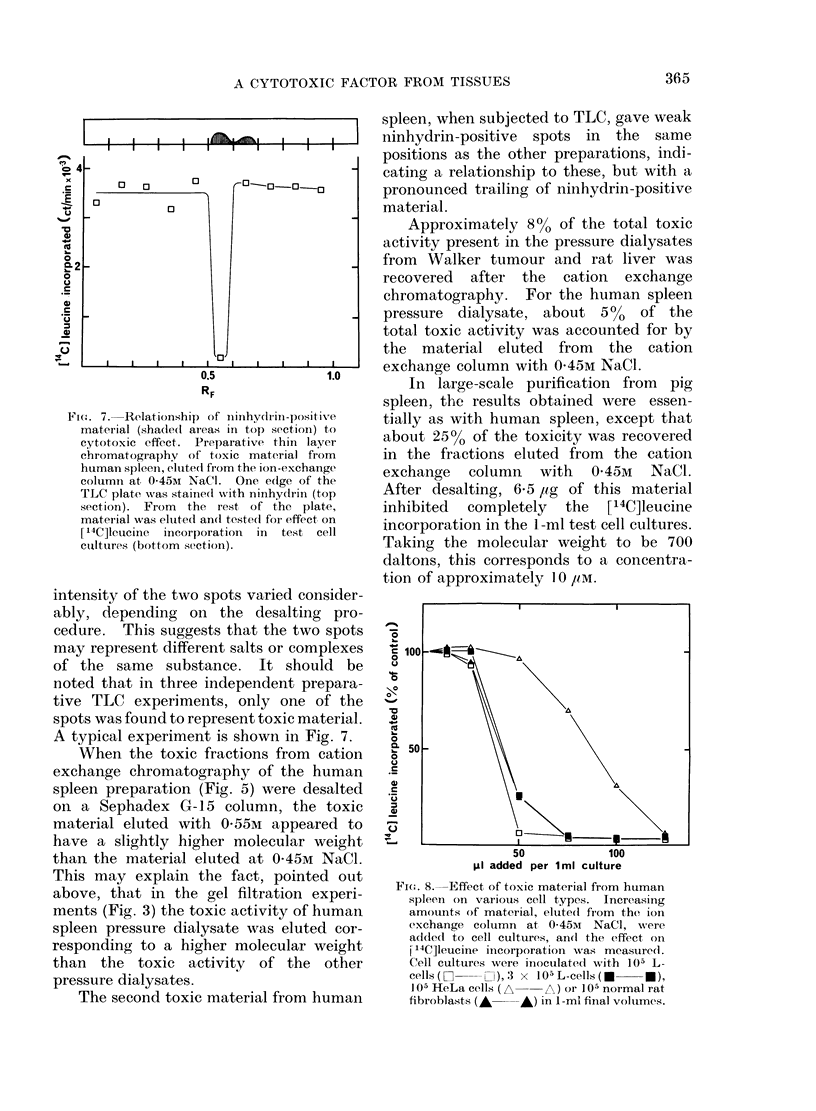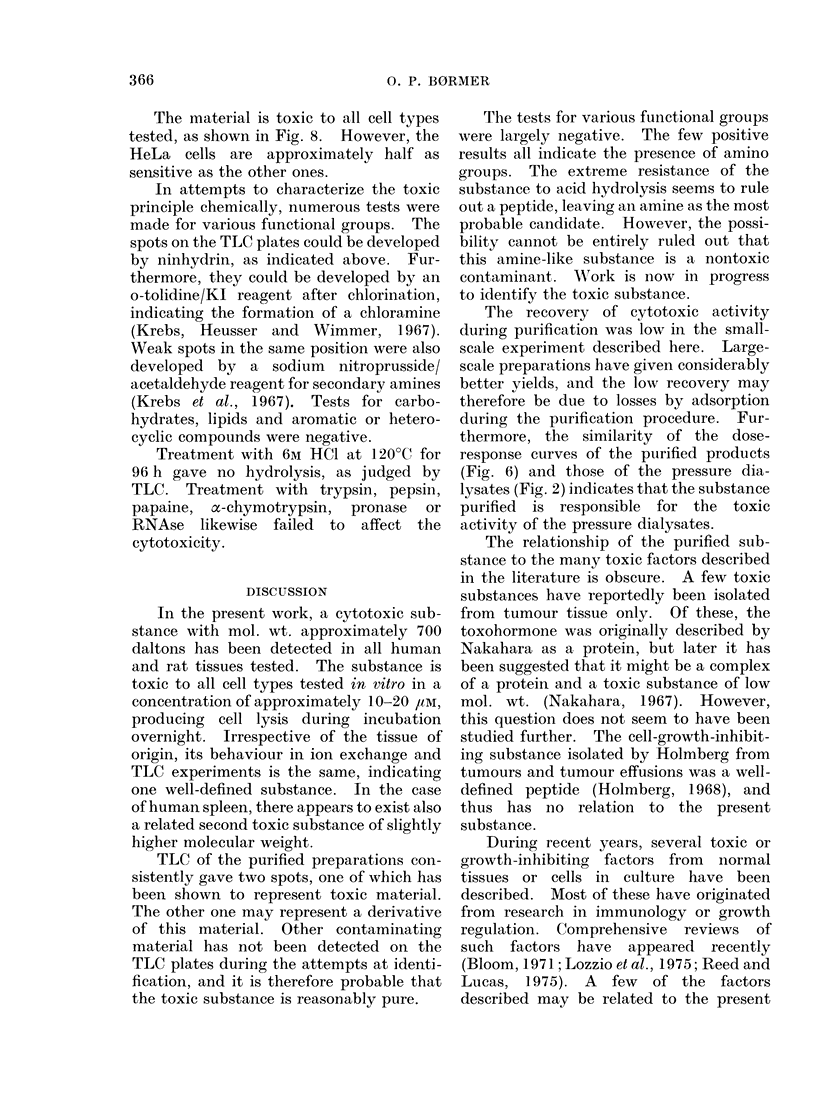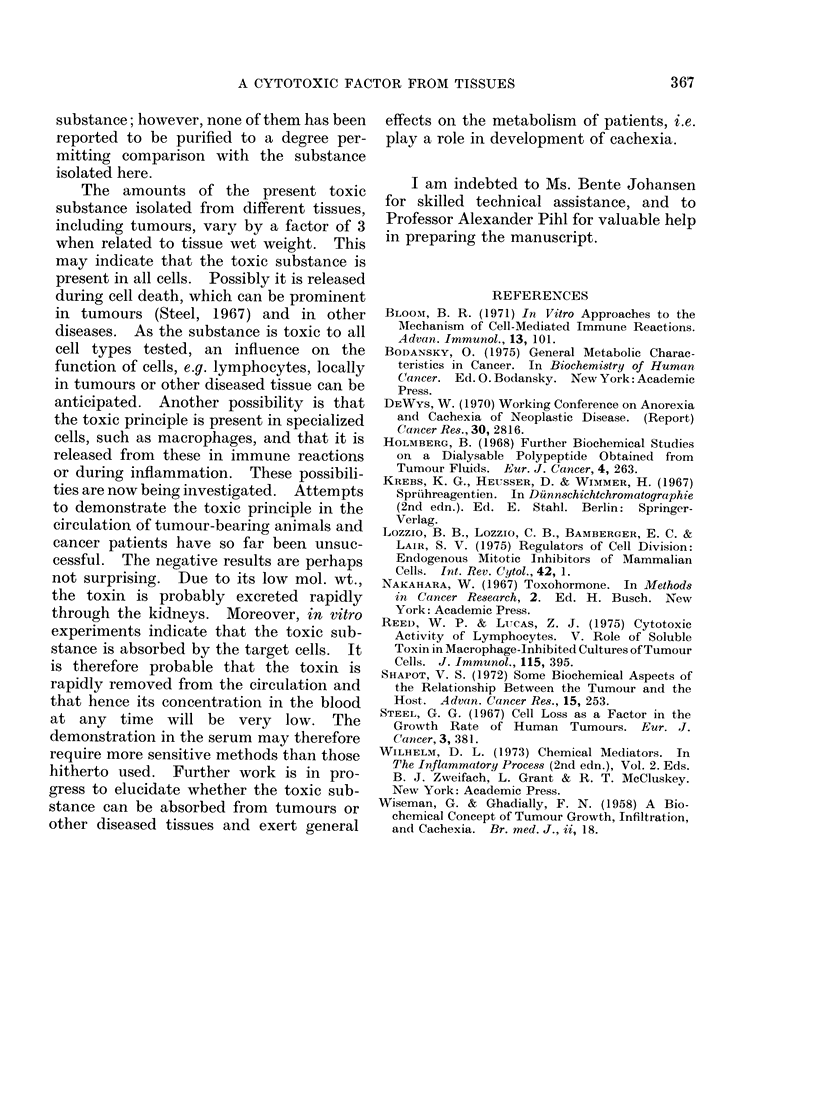# Purification of a cytotoxic factor from human and rat tissues.

**DOI:** 10.1038/bjc.1976.178

**Published:** 1976-10

**Authors:** O. P. Bormer

## Abstract

Human and rat normal tissues and tumours have been studied for the presence of toxic substances, possibly of importance in the development of cachexia in patients with cancer and other chronic diseases. The toxic effect of tissue extracts was gauged by measuring the inhibition of growth of mouse L-cells in 1-ml cultures, as revealed by reduced incorporation of [14C]leucine into cell protein. A common cytotoxic substance of mol. wt. approximately 700 daltons was isolated from all rat and human tissues tested, including tumours. The isolation procedure involved tissue homogenization, followed by pressure dialysis, gel filtration of concentrated pressure dialysates, cation exchange chromatography, and thin layer chromatography. Amounts isolated from different tissues varied by a factor of 3. The purified substance reacted with ninhydrin and a few other reagents for amino groups. It was completely resistant to acid and enzymatic hydrolysis. The evidence thus suggests that the substance is an amine. It is toxic to L-cells, HeLa cells and normal rat fibroblasts in concentrations of 10-20 muM, producing cell death and lysis during incubation overnight.


*Br. J. Cancer* (1976) **34**, 359

### PURIFICATION OF A CYTOTOXIC FACTOR FROM HUMAN AND RAT TISSUES

O. P. BØRMER*

*From Norsk Hydro's Institute for Cancer Research, The Norwegian Radium Hospital, Montebello, Oslo 3, Norway*

Received 30 March 1976 Accepted 17 June 1976

**Summary.—Human and rat normal tissues and tumours have been studied for the presence of toxic substances, possibly of importance in the development of cachexia in patients with cancer and other chronic diseases. The toxic effect of tissue extracts was gauged by measuring the inhibition of growth of mouse L-cells in 1-ml cultures, as revealed by reduced incorporation of [$^{14}$C]leucine into cell protein.**

**A common cytotoxic substance of mol. wt. approximately 700 daltons was isolated from all rat and human tissues tested, including tumours. The isolation procedure involved tissue homogenization, followed by pressure dialysis, gel filtration of concentrated pressure dialysates, cation exchange chromatography, and thin layer chromatography. Amounts isolated from different tissues varied by a factor of 3. The purified substance reacted with ninhydrin and a few other reagents for amino groups. It was completely resistant to acid and enzymatic hydrolysis. The evidence thus suggests that the substance is an amine. It is toxic to L-cells, HeLa cells and normal rat fibroblasts in concentrations of 10–20 μM, producing cell death and lysis during incubation overnight.**

CACHEXIA is frequently seen in patients with advanced cancer (DeWys, 1970; Bodansky, 1975). In a less pronounced form, the condition may also be seen in patients in whom no organ or function of major importance is directly impaired by the tumour or its metastases (Shapot, 1972). Although the problem of cachexia is of great importance in the care and treatment of the cancer patient, the underlying metabolic disturbances have received relatively little attention, and they are largely still unexplained. However, several theories have been put forward. It has been suggested that the tumour may have greater affinity than normal tissue for glucose and amino acids, and thus act as a metabolic trap for these important nutrient factors (Wiseman and Ghadially, 1958; DeWys, 1970; Shapot, 1972). Some tumours produce hormones or hormone-like substances which can have profound influence on the metabolism of the host (DeWys, 1970). It has also been suggested that malignant tumours may generate toxic products with general effect on the host, and toxic substances have in fact been isolated from some malignant tumours and tumour effusions (Nakahara, 1967; Holmberg, 1968). However, these theories fail to account for the fact that cachexia may also develop in patients with other grave diseases of some duration (DeWys, 1970).

Evidence has accumulated that toxic substances may be produced during inflammation (Wilhelm, 1973) and immune response (Bloom, 1971; Reed and Lucas, 1975). Such processes are activated both in neoplastic and non-neoplastic disease. A study was therefore initiated of the presence of toxic factors in malignant as well as non-malignant tissues. Here we wish to report on the purification of a

* Fellow of the Norwegian Cancer Society.

substance which is toxic to cells in culture and which is present in all rat and human tissues tested.

### MATERIALS AND METHODS

*Cell cultures.*—Eagle's Minimum Essential Medium with Earle's salts (GIBCO Biocult Ltd., Glasgow, Scotland), 10% calf serum (GIBCO Biocult), 100 μg streptomycin/ml and 100 iu penicillin/ml was used throughout. Cell cultures were incubated at 37°C in an atmosphere of 5% $CO_2$ in air.

Mouse L-cells of clone 929 were maintained as monolayer cultures in plastic culture flasks (Falcon, Oxnard, California, U.S.A.). For test cultures, the cells were trypsinized with a 0·25% trypsin solution, washed once in cell culture medium and adjusted to the desired density in medium.

Unless otherwise stated, test cultures were inoculated with $10^5$ L-cells in a total volume of 1 ml. Included in the 1 ml volume were 0·1 or 0·2 ml test solution, 100 μg gentamycin (Garamycin, Schering Corporation, Kenilworth, New Jersey, U.S.A.) and 0·05 or 0·1 μCi L[U-$^{14}$C]leucine (324 mCi/mmol) (The Radiochemical Centre, Amersham, England). 1·6 × 10-cm plastic culture tubes (Falcon) were used for all test cultures.

In one experiment, HeLa cells and normal rat fibroblasts were used. The HeLa cells were maintained and treated as described for the L-cells, while normal rat fibroblasts were obtained by trypsinization of minced skin and subcutaneous tissue from newborn rats and maintained in culture for 10 days before use in experiments. Experimental conditions with these cell types were as described for the L-cells.

All test cultures were incubated overnight (15–20 h), and their morphological appearances were evaluated microscopically before harvesting. Bacterial contamination was monitored by microscopy, followed by plating of media aliquots from suspected cultures. In no instance was bacterial growth detected in test cell cultures.

The cultures were washed twice by adding 3 ml phosphate-buffered saline (PBS: 0·14M sodium chloride, 0·01M sodium phosphate, pH 7·5) centrifuging at 700 *g* for 5 min and drawing off the supernatant by suction. The cells were dissolved in 0·1M KOH, followed by precipitation in 10% (w/v) trichloroacetic acid. The precipitates were collected on 25-mm glass fibre filters and washed 3 times with 5 ml 10% (w/v) trichloroacetic acid. The filters were transferred to counting vials and dried for 1 h at 95°C. Five ml of toluene-based scintillation liquid was added, and the radioactivity was measured in a liquid scintillation spectrometer with 70% efficiency. Counting time was chosen to give less than 2% counting error for the uninhibited cultures. The resultant coefficient of variation for the incorporation of [$^{14}$C]leucine into protein in a series of control cultures was less than 3%.

*Tissues.*—Walker 256 carcinosarcoma was maintained by transplantation to the axillary region of 6–8-week-old Wistar rats. Tumours were allowed to grow for 14 to 16 days, reaching a diameter of about 2 cm. Normal rat tissues were obtained from animals of the same litters as the tumour-bearing ones.

The rats were killed by cervical dislocation under light ether anaesthesia, and the tumours and normal tissues were removed aseptically. Small necrotic areas were removed from the tumours: those showing more than minimal necrosis were discarded. Human spleen tissue was obtained from patients with Hodgkin's disease undergoing diagnostic laparotomy with splenectomy. Only tissues from histologically normal spleens were used. All tissues were stored under sterile conditions at −20°C until further processing.

*Preparation of tissue extracts.*—40–60 g of each tissue was minced with scissors and homogenized for 120–180 s (30 s per 10 g of tissue) in 2 volumes of PBS at 0°C with an X-1020 homogenizer at 25,000 rev/min (Internationale Laboratoriums-Apparate GmbH, Dottingen, W. Germany). The homogenates were centrifuged at 27,000 *g* for 30 min at 4°C. The precipitates were discarded. Aliquots of the supernatants were frozen and stored at −20°C for later testing. All operations were done aseptically.

*Pressure dialysis.*—The remaining supernatants were pressure dialyzed with a 1 kg/cm$^2$ $N_2$ pressure through 30-cm lengths of 1-cm dialysis tubing (Union Carbide Corporation, Chicago, Illinois, U.S.A.). The tubing had previously been boiled and stored in a buffer with 25 mM $NaHCO_3$, 10 mM $Na_2B_4O_7$ and 10 mM EDTA, pH 9, and was extensively rinsed with distilled water before use. Aliquots of the pressure dialysates were frozen at −20°C for later testing. 45 ml of each pressure dialysate was freeze-dried. The residues in the tubings were discarded.

*Gel filtration.*—Each of the freeze-dried pressure dialysates was dissolved in 2·25 ml water (1/20 of their original volume), giving total volumes of 2·65 to 2·85 ml. Precipitates were removed by centrifugation. Gel filtration of 2·3 ml of each concentrated pressure dialysate was performed on a column of Sephadex G-15 (Pharmacia, Uppsala, Sweden) as described in the legend to Fig. 3, after the addition of 1 mg Blue Dextran 2000 (Pharmacia) and 0·5 $\mu$Ci [L 4,5-$^3$H]leucine (Radiochemical Centre). The void volume was determined by spectrophotometry at 640 nm of the Blue Dextran 2000. The elution volume of [$^3$H]leucine was determined by counting the $^3$H activity in 50 $\mu$l of each fraction. The elution volume of low molecular weight salts from the concentrated pressure dialysates was determined by measuring conductivity with a CDM3 conductivity meter (Radiometer, Copenhagen, Denmark). The fractions indicated by brackets in Fig. 3 were pooled and diluted to 20 ml with PBS. Six ml of each preparation was frozen and stored at −20°C for later testing.

*Cation exchange chromatography.*—The remaining 14 ml of the pooled fractions from gel filtration was loaded on to columns of carboxymethyl cellulose (CM-52, Whatman Biochemicals Ltd., Maidstone, England) equilibrated with PBS. Elution was performed with NaCl gradients as described in the legend to Fig. 5. The NaCl concentration of the collected fractions was estimated from the measured conductivity, using a standard curve. The fractions in the range from 0·40M to 0·48M NaCl were pooled, as indicated by brackets in Fig. 5. The pooled fractions were freeze-dried, dissolved in a minimal volume of water, and desalted on a column of Sephadex G-15 in water. A sample of each fraction obtained from the desalting column was applied to a thin layer chromatography (TLC) plate, dried and stained with ninhydrin reagent as described in the section on analytical TLC below. Fractions giving a positive ninhydrin reaction were pooled. Small volumes were removed for analytical TLC. The remaining material was freeze-dried, redissolved in 2 ml PBS, frozen and stored at −20°C for later testing.

*Titration of toxicity.*—In one large experiment, the frozen samples from each stage in the purification procedure were thawed, and dilutions made with PBS in small increments. From each dilution, 0·2 ml was transferred to test cell cultures.

*Human spleen.*—As seen from Fig. 5, cation exchange chromatography of this preparation yielded two cytotoxic components eluted at 0·45M and 0·55M NaCl. To evaluate the relationship between these, 100 g of human spleen tissue was processed as described above, except that no samples were removed for later testing. The two toxic preparations from the CM-52 column were concentrated by freeze-drying and desalted on a Sephadex G-15 column. From each preparation, one part of the desalted material was used in TLC experiments, while the other one was freeze-dried and redissolved in PBS for cell experiments.

*Analytical TLC.*—Aliquots of the desalted material, in amounts approximately inversely proportional to their toxicity, were applied to a cellulose TLC plate (TLC Ready Plate G-1440, Carl Schleicher & Schüll, Dassel, W. Germany). The plate was developed with a mixture of 80 parts 96% ethanol and 20 parts 25% (w/v) ammonia. After drying, the plate was sprayed with a solution of 0·3% (w/v) ninhydrin and 3% (v/v) acetic acid in butanol-(1), and heated for 5 min at 110°C.

*Preparative TLC.*—Material from human spleen, eluted from the CM-52 column at 0·45M NaCl, was applied as a line on a cellulose TLC plate. The amount applied was 10 times the minimum inhibitory dose for the test cell cultures. The TLC plate was developed as described in the preceding section. One edge of the plate was stained with ninhydrin, while the cellulose was removed in 10 fractions from the rest of the plate. Each fraction was eluted with 2 × 2 ml 5% (w/v) ammonia The eluates were freeze-dried, dissolved in 0·2 ml PBS, and transferred to test cell cultures.

*Effect on various cells and cell concentrations.*—Material from human spleen, eluted from the CM-52 column at 0·45M NaCl, was diluted with PBS in increments. From each dilution, 0·2 ml was transferred to duplicate cell cultures as described in the legend to Fig. 8.

### RESULTS

In the present study, toxic factors were looked for by testing the ability of tissue extracts to inhibit the growth of cells in culture. Rather than expose the cells to the agent to be tested for a period, remove the agent and then measure cell growth, it was decided to measure cell growth in the presence of the tissue extracts. This direct method is simpler and seems to be more relevant to the metabolic situation of the cachectic patient. The incorporation of [$^{14}$C]leucine into cell protein was used as a general measure of cell growth; the results are therefore given as counts/min incorporated per culture, and conclusions are based on comparison with the control cultures. To facilitate the experiments, test material was added to the culture tubes shortly after the addition of the cell suspension, not waiting for the cells to attach as a monolayer. In control experiments, this rapid technique yielded the same results as adding the test material to established monolayer cultures.

During the purification procedure, great care was taken to avoid bacterial contamination. However, with the large number of manipulations involved, the use of antibiotics in fairly high concentrations in the test cell cultures was deemed necessary. In control experiments, the antibiotics in the concentrations used had no detectable effect on cell protein synthesis or on the inhibitory properties of the toxic principle.

The results in Fig. 1 show that the supernatants from different tissues inhibited the incorporation of [$^{14}$C]leucine in the test cells to a varying extent and with widely differing dose-response curves. On dialysis against large volumes of PBS, the inhibitory effect was lost (data not shown). The pressure dialysates from three different tissues also showed marked inhibitory effects, indicating a low molecular weight of the inhibitory principle(s). As all macromolecules were removed during pressure dialysis it was irrelevant to correlate toxicity to any of the commonly used parameters, *e.g.* amount of protein, during the further purification of

FIG. 1.—Dose-response curves for the effect of supernatants of homogenates from different tissues on [$^{14}$C]leucine incorporation into protein of L-cells in culture. Each culture initially contained $10^5$ cells in a total volume of 1 ml, including the test solution and 0·1 $\mu$Ci [$^{14}$C]leucine. The amount of radioactivity incorporated was measured after 18 h incubation at 37°C. Further details are given in the text. ●——● normal rat abdominal wall; □——□ human spleen; ○——○ normal rat liver; ×——× Walker 256 tumour.

FIG. 2.—Dose-response curves for the effect of pressure dialysates from supernatants on [$^{14}$C]leucine incorporation into protein of L-cells in culture. Cell cultures were as described in legend to Fig. 1. ●——● normal rat abdominal wall; □——□ human spleen; ○——○ normal rat liver; ×——× Walker 256 tumour.

the toxic principle(s). Instead, all procedures, volumes and dilutions were strictly standardized, thereby permitting comparison of the different tissue preparations, and the amount of material added to test-cell cultures is consequently expressed in terms of volumes. Fig. 2 shows that the dose-response curves of the pressure dialysates from rat liver, Walker 256 tumour and human spleen were fairly similar, while the abdominal wall pressure dialysate had approximately half the inhibitory effect of the other ones. The more uniform toxicity of the pressure dialysates compared to the corresponding supernatants suggests that high-molecular-weight interfering substances, possibly present in different amounts in the different preparations, were removed during pressure dialysis. After incubation of test cell cultures overnight, microscopic inspection showed cell lysis in the completely inhibited cultures, while the cells in the control and uninhibited cultures formed uniform monolayers.

During gel filtration of the concentrated pressure dialysates on Sephadex G-15, the cytotoxic activity of the Walker tumour and rat liver preparations was eluted corresponding to a mol. wt. of about 700 daltons (Fig. 3). The toxic activity from human spleen appeared in fractions corresponding to a slightly higher molecular weight. The normal abdominal wall preparation showed no inhibitory effect in this system. However, when more of this preparation was added, a moderate inhibition of the test cells was found (Fig. 4).

In order to rule out the possibility that the reduced cellular incorporation of [$^{14}$C]leucine into protein was due to

FIG. 3.—Gel filtration of the cytotoxic factor(s) from concentrated pressure dialysates. A 2·6 × 35-cm column of Sephadex G-15 was eluted with PBS at 1·2 ml/min, and the eluate collected in 2·4-ml. fractions. From each fraction, 0·1 ml was transferred to test cell cultures, and the effect on [$^{14}$C]leucine incorporation into protein was measured (ordinates). Cell cultures were as described in legend to Fig. 1. *a*, void volume; *b*, peak of [$^{3}$H]leucine, added as a marker for the elution of endogenous leucine from tissues; *c*, peak of conductivity. Fractions indicated by brackets were pooled.

FIG. 4.—Dose-response curves for the effect of pooled fractions from gel filtration (Fig. 3) on [$^{14}$C]leucine incorporation into protein of L-cells in culture. Cell cultures were as described in legend to Fig. 1. ●——● normal rat abdominal wall; □——□ human spleen; ○——○ normal rat liver; ×——× Walker 256 tumour.

FIG. 5.—Cation exchange chromatography of the cytotoxic factor(s) from the pooled fractions after gel filtration (Fig. 3). 1·6 × 12-cm columns of carboxymethylcellulose equilibrated with PBS were loaded with the pooled fractions, and elution was performed with linear gradients of NaCl in 10 mM sodium phosphate, pH 7·5, at 0·6 ml/min. Two-ml fractions were collected (abscissae). From each fraction, 0·1 ml was transferred to test cell cultures, and the effect on [$^{14}$C] leucine incorporation was measured (left ordinates). Cell cultures were as described in legend to Fig. 1. · · · · · · NaCl concentration (right ordinates). Fractions indicated by brackets were pooled and desalted.

dilution by unlabelled leucine from tissues, [$^{3}$H]leucine was used as a marker for the elution of unlabelled leucine in the gel filtration experiments. The cytotoxic effect was eluted well separated from leucine (Fig. 3). In a second control experiment, [$^{3}$H]thymidine was added as a marker. The cytotoxic effect was eluted well separated from the thymidine (data not shown), ruling out the possibility that the inhibition of cell growth could be due to the presence of thymidine in the extracts.

In the cation exchange chromatography, the cytotoxic activity was eluted with 0·45M NaCl in the case of the Walker tumour, rat liver and human spleen preparations (Fig. 5). The human spleen in addition gave a second peak of toxic activity which was eluted at 0·55M NaCl. Again, the inhibitory effect of the abdominal wall preparation was not detected in the first instance. However, when larger amounts of the material eluted with 0·45M NaCl was added to the test cell cultures, a slight inhibition was found even with this preparation (Fig. 6). It is seen that the other preparations gave complete inhibition of protein synthesis, and microscopic examination after incubation overnight showed cell lysis in the inhibited cultures.

The material eluted with 0·45M NaCl was desalted and subjected to analytical TLC. The same pattern was found for all preparations, including the one from the abdominal wall, with two partly confluencing ninhydrin-positive spots with $R_F$ values of approximately 0·55 and 0·65, as indicated in the top section of Fig. 7. In different experiments, the relative

FIG. 6.—Dose-response curves for the effect of pooled and desalted fractions from cation exchange chromatography (Fig. 5) on [$^{14}$C] leucine incorporation into protein of L-cells in culture. Cell cultures were as described in legend to Fig. 1. ●——● normal rat abdominal wall; □——□ human spleen; ○——○ normal rat liver; ×——× Walker 256 tumour.

FIG. 7.—Relationship of ninhydrin-positive material (shaded areas in top section) to cytotoxic effect. Preparative thin layer chromatography of toxic material from human spleen, eluted from the ion-exchange column at 0·45M NaCl. One edge of the TLC plate was stained with ninhydrin (top section). From the rest of the plate, material was eluted and tested for effect on [$^{14}$C]leucine incorporation in test cell cultures (bottom section).

intensity of the two spots varied considerably, depending on the desalting procedure. This suggests that the two spots may represent different salts or complexes of the same substance. It should be noted that in three independent preparative TLC experiments, only one of the spots was found to represent toxic material. A typical experiment is shown in Fig. 7.

When the toxic fractions from cation exchange chromatography of the human spleen preparation (Fig. 5) were desalted on a Sephadex G-15 column, the toxic material eluted with 0·55M appeared to have a slightly higher molecular weight than the material eluted at 0·45M NaCl. This may explain the fact, pointed out above, that in the gel filtration experiments (Fig. 3) the toxic activity of human spleen pressure dialysate was eluted corresponding to a higher molecular weight than the toxic activity of the other pressure dialysates.

The second toxic material from human spleen, when subjected to TLC, gave weak ninhydrin-positive spots in the same positions as the other preparations, indicating a relationship to these, but with a pronounced trailing of ninhydrin-positive material.

Approximately 8% of the total toxic activity present in the pressure dialysates from Walker tumour and rat liver was recovered after the cation exchange chromatography. For the human spleen pressure dialysate, about 5% of the total toxic activity was accounted for by the material eluted from the cation exchange column with 0·45M NaCl.

In large-scale purification from pig spleen, the results obtained were essentially as with human spleen, except that about 25% of the toxicity was recovered in the fractions eluted from the cation exchange column with 0·45M NaCl. After desalting, 6·5 $\mu$g of this material inhibited completely the [$^{14}$C]leucine incorporation in the 1-ml test cell cultures. Taking the molecular weight to be 700 daltons, this corresponds to a concentration of approximately 10 $\mu$M.

FIG. 8.—Effect of toxic material from human spleen on various cell types. Increasing amounts of material, eluted from the ion exchange column at 0·45M NaCl, were added to cell cultures, and the effect on [$^{14}$C]leucine incorporation was measured. Cell cultures were inoculated with $10^5$ L-cells (□——□), 3 × $10^5$ L-cells (■——■), $10^5$ HeLa cells (△——△) or $10^5$ normal rat fibroblasts (▲——▲) in 1-ml final volumes.

The material is toxic to all cell types tested, as shown in Fig. 8. However, the HeLa cells are approximately half as sensitive as the other ones.

In attempts to characterize the toxic principle chemically, numerous tests were made for various functional groups. The spots on the TLC plates could be developed by ninhydrin, as indicated above. Furthermore, they could be developed by an o-tolidine/KI reagent after chlorination, indicating the formation of a chloramine (Krebs, Heusser and Wimmer, 1967). Weak spots in the same position were also developed by a sodium nitroprusside/acetaldehyde reagent for secondary amines (Krebs *et al.*, 1967). Tests for carbohydrates, lipids and aromatic or heterocyclic compounds were negative.

Treatment with 6M HCl at 120°C for 96 h gave no hydrolysis, as judged by TLC. Treatment with trypsin, pepsin, papaine, $\alpha$-chymotrypsin, pronase or RNAse likewise failed to affect the cytotoxicity.

### DISCUSSION

In the present work, a cytotoxic substance with mol. wt. approximately 700 daltons has been detected in all human and rat tissues tested. The substance is toxic to all cell types tested *in vitro* in a concentration of approximately 10–20 $\mu$M, producing cell lysis during incubation overnight. Irrespective of the tissue of origin, its behaviour in ion exchange and TLC experiments is the same, indicating one well-defined substance. In the case of human spleen, there appears to exist also a related second toxic substance of slightly higher molecular weight.

TLC of the purified preparations consistently gave two spots, one of which has been shown to represent toxic material. The other one may represent a derivative of this material. Other contaminating material has not been detected on the TLC plates during the attempts at identification, and it is therefore probable that the toxic substance is reasonably pure.

The tests for various functional groups were largely negative. The few positive results all indicate the presence of amino groups. The extreme resistance of the substance to acid hydrolysis seems to rule out a peptide, leaving an amine as the most probable candidate. However, the possibility cannot be entirely ruled out that this amine-like substance is a nontoxic contaminant. Work is now in progress to identify the toxic substance.

The recovery of cytotoxic activity during purification was low in the small-scale experiment described here. Large-scale preparations have given considerably better yields, and the low recovery may therefore be due to losses by adsorption during the purification procedure. Furthermore, the similarity of the dose-response curves of the purified products (Fig. 6) and those of the pressure dialysates (Fig. 2) indicates that the substance purified is responsible for the toxic activity of the pressure dialysates.

The relationship of the purified substance to the many toxic factors described in the literature is obscure. A few toxic substances have reportedly been isolated from tumour tissue only. Of these, the toxohormone was originally described by Nakahara as a protein, but later it has been suggested that it might be a complex of a protein and a toxic substance of low mol. wt. (Nakahara, 1967). However, this question does not seem to have been studied further. The cell-growth-inhibiting substance isolated by Holmberg from tumours and tumour effusions was a well-defined peptide (Holmberg, 1968), and thus has no relation to the present substance.

During recent years, several toxic or growth-inhibiting factors from normal tissues or cells in culture have been described. Most of these have originated from research in immunology or growth regulation. Comprehensive reviews of such factors have appeared recently (Bloom, 1971; Lozzio *et al.*, 1975; Reed and Lucas, 1975). A few of the factors described may be related to the present substance; however, none of them has been reported to be purified to a degree permitting comparison with the substance isolated here.

The amounts of the present toxic substance isolated from different tissues, including tumours, vary by a factor of 3 when related to tissue wet weight. This may indicate that the toxic substance is present in all cells. Possibly it is released during cell death, which can be prominent in tumours (Steel, 1967) and in other diseases. As the substance is toxic to all cell types tested, an influence on the function of cells, *e.g.* lymphocytes, locally in tumours or other diseased tissue can be anticipated. Another possibility is that the toxic principle is present in specialized cells, such as macrophages, and that it is released from these in immune reactions or during inflammation. These possibilities are now being investigated. Attempts to demonstrate the toxic principle in the circulation of tumour-bearing animals and cancer patients have so far been unsuccessful. The negative results are perhaps not surprising. Due to its low mol. wt., the toxin is probably excreted rapidly through the kidneys. Moreover, *in vitro* experiments indicate that the toxic substance is absorbed by the target cells. It is therefore probable that the toxin is rapidly removed from the circulation and that hence its concentration in the blood at any time will be very low. The demonstration in the serum may therefore require more sensitive methods than those hitherto used. Further work is in progress to elucidate whether the toxic substance can be absorbed from tumours or other diseased tissues and exert general effects on the metabolism of patients, *i.e.* play a role in development of cachexia.

I am indebted to Ms. Bente Johansen for skilled technical assistance, and to Professor Alexander Pihl for valuable help in preparing the manuscript.

### REFERENCES

Bloom, B. R. (1971) *In Vitro* Approaches to the Mechanism of Cell-Mediated Immune Reactions. *Advan. Immunol.*, **13**, 101.

Bodansky, O. (1975) General Metabolic Characteristics in Cancer. In *Biochemistry of Human Cancer*. Ed. O. Bodansky. New York: Academic Press.

DeWys, W. (1970) Working Conference on Anorexia and Cachexia of Neoplastic Disease. (Report) *Cancer Res.*, **30**, 2816.

Holmberg, B. (1968) Further Biochemical Studies on a Dialysable Polypeptide Obtained from Tumour Fluids. *Eur. J. Cancer*, **4**, 263.

Krebs, K. G., Heusser, D. & Wimmer, H. (1967) Sprühreagentien. In *Dünnschichtchromatographie* (2nd edn.). Ed. E. Stahl. Berlin: Springer-Verlag.

Lozzio, B. B., Lozzio, C. B., Bamberger, E. C. & Lair, S. V. (1975) Regulators of Cell Division: Endogenous Mitotic Inhibitors of Mammalian Cells. *Int. Rev. Cytol.*, **42**, 1.

Nakahara, W. (1967) Toxohormone. In *Methods in Cancer Research*, **2**. Ed. H. Busch. New York: Academic Press.

Reed, W. P. & Lucas, Z. J. (1975) Cytotoxic Activity of Lymphocytes. V. Role of Soluble Toxin in Macrophage-Inhibited Cultures of Tumour Cells. *J. Immunol.*, **115**, 395.

Shapot, V. S. (1972) Some Biochemical Aspects of the Relationship Between the Tumour and the Host. *Advan. Cancer Res.*, **15**, 253.

Steel, G. G. (1967) Cell Loss as a Factor in the Growth Rate of Human Tumours. *Eur. J. Cancer*, **3**, 381.

Wilhelm, D. L. (1973) Chemical Mediators. In *The Inflammatory Process* (2nd edn.), Vol. 2. Eds. B. J. Zweifach, L. Grant & R. T. McCluskey. New York: Academic Press.

Wiseman, G. & Ghadially, F. N. (1958) A Biochemical Concept of Tumour Growth, Infiltration, and Cachexia. *Br. med. J.*, *ii*, 18.